# Evaluation of behavioural and antioxidant activity of *Cytisus scoparius *Link in rats exposed to chronic unpredictable mild stress

**DOI:** 10.1186/1472-6882-8-15

**Published:** 2008-04-24

**Authors:** Jayabalan Nirmal, Chidambaram Saravana Babu, Thanukrishnan Harisudhan, Muthiah Ramanathan

**Affiliations:** 1 Neuropharmacology Laboratory, Department of Pharmacology, J.S.S. College of Pharmacy, Rocklands, Ootacamund, TN 643 001, India

## Abstract

**Background:**

Various human diseases have oxidative stress as one of their component. Many herbs have been reported to exhibit properties that combat oxidative stress through their active constituents such as flavonoids, tannins, phenolic compounds etc. *Cytisus scoparius *(CS) Link, (Family: Leguminosae), also called *Sarothamnus scoparius*, has been shown in *invitro *experiments to be endowed with anti-diabetic, hypnotic and sedative and antioxidant activity. Therefore this study was carried out to evaluate CS for its anxiolytic, antidepressant and anti-oxidant activity in stressed rats.

**Methods:**

60% methanolic extract of CS was quantified for phenolic content by Folin-Ciocalteau's method. Chronic unpredictable mild stress (CMS) was employed to induce stress in rats. CS (125 and 250 mg/kg, p.o) and diazepam (DZM) (2 mg/kg, p.o) was administered during the 21 day stress exposure period. Anxiolytic and antidepressant activities of CS were assessed in open field exploratory and behavioural despair paradigms, respectively. Plasma glucose and total lipids; endogenous antioxidant enzymes such as superoxide dismutase (SOD), catalase (CAT); non-enzymic-ascorbic acid and thiobarbituric acid reactive substances (TBARS) levels were measured in brain, kidneys and adrenals using standard protocols to assess the effect of CS.

**Results:**

Total phenolic content of CS was found to be 8.54 ± 0.16% w/w. CMS produced anxiogenic and depressive behaviour in experimental rats with metabolic disturbance. Significant decrease in SOD, CAT levels and increase in lipid peroxidation level was observed in stressed rats. CS administration for 21 days during stress exposure significantly increased the ambulatory behaviour and decreased the freezing time in open field behaviour. In behavioural despair test no significant alteration in the immobility period was observed. CS also improved SOD, CAT, and ascorbic acid level and controlled the lipid peroxidation in different tissues.

**Conclusion:**

CS possesses anti-stress and moderate anxiolytic activity which may be due, in part, to its antioxidant effect that might warrant further studies.

## Background

According to the World Health report, approximately 450 million people suffer from a mental or behavioural disorder [1]. This amounts to 12.3% of the global burden of disease, and predicted to raise upto 15% by 2020 [2]. Stress is a state of threatened homeostasis provoked by psychological, physiological or environmental stressors [3]. Stressor is a stimulus either internal or external, which activates the hypothalamic pituitary adrenal axis and the sympathetic nervous system resulting in a physiological change [4]. Stressful conditions can precipitate anxiety and depression, which can lead to excessive production of free radicals which in turn results in oxidative stress, an imbalance in the oxidant/antioxidant system. Currently different therapeutic regimens are employed to treat anxiety and depressive disorders; but their clinical uses are limited by their side effects such as psychomotor impairment, potentiation of other central depressant drugs and dependence liability. In the search for new therapeutic products for the treatment of neurological disorders – medicinal plant research has also contributed significantly by demonstrating pharmacological effectiveness of different herbs in various animal models [5].

*Cytisus scoparius *Link., (Family: Leguminosae) also called as *Sarothamnus scoparius *is a popular herb in Ayurveda. CS species is commonly available in Nilgiris biosphere of Tamil Nadu, India. Ethnomedical information states that this herb is used as diuretics, hypnotics, sedative [6]. Experimental reports indicated that CS possessed anti-diabetic [7], hepatoprotective [8], antispasmodic, hypotensive and estrogenic effect [9]. The pharmacological activity of CS was attributed due to the chemical constituents 6-O-acetyl scoparian [10], flavonals like rutin, quercitin, isorhamnetin, quercitrin and kaempferol [11] and isoflavones namely genistein and sarothamnoside [12]. Alkaloids like spartein, sarothamine and lupamine were also reported to be present in CS [13].

Conditions of stress precipitated glucose intolerance, immuno-suppression, behavioural depression, cognitive deficits and male sexual dysfunction in rats. Administration of *Withania somnifera *during the stress period alleviated these alterations. It was postulated that, at least in some instances, the beneficial effect of *Withania somnifera *was due to its anti-oxidative stress action [14,15]. Like wise the antioxidant property of CS in *in vitro *and *in vivo *models was attributed to its phenolic content [16,17]. This formed the basis for the present investigation which aimed to investigate the potential role of CS in reversing the behavioural and biochemical alterations in CMS rats.

## Methods

### Chemicals

All chemicals and solvents used were of analytical grade and were obtained from SISCO Research Laboratories, Mumbai, India.

### Plant material

CS collected from Nilgiris Biosphere, Tamil Nadu, India was authenticated by Botanist, Dr. S. Rajan, Survey of Indian medicinal plants, Govt. Arts College, Ootacamund. For future references voucher specimen of CS was preserved as herbarium in department of Pharmacognosy, JSS College of Pharmacy, Ooty, India.

### Preparation of extract

Shade dried aerial parts of CS was coarsely powdered and macerated with 60% methanol at room temperature for 72 h. The filtrate was dried in a rotary vacuum evaporator under reduced pressure at 50°C. The extract was stored in desiccator for future use.

### Estimation of total phenolic content

Total phenolic content of CS was estimated by employing Follin-Ciocalteau method [18] as stated here. Extract solution of 0.1 ml CS (containing 1000 μg) was taken in a volumetric flask and diluted with distilled water to 46 ml. About 1 ml of Folin – Ciocalteu reagent was added to the contents of the flask and mixed thoroughly. After 3 min, 3 ml of Na_2_CO_3 _(2%) was added and the mixture was allowed to stand for 2 h with intermittent shaking. The absorbance was measured at 760 nm. Phenolic content was calculated using pyrocatechol as standard.

### Animals

Thirty adult male Wistar strain rats (160–175 g) were procured from the central animal facility and divided into five groups of six animals each. They were housed in colony cages at an ambient temperature of 25 ± 2°C and 40–65% relative humidity, with 12-h light: dark cycle. The animals had free access to standard pellet chow and drinking water. Institutional Animal Ethics Committee (IAEC) approved the study and all the experiments were carried out by following the guidelines of CPCSEA, India.

### Groups and treatment

**Figure 1:**
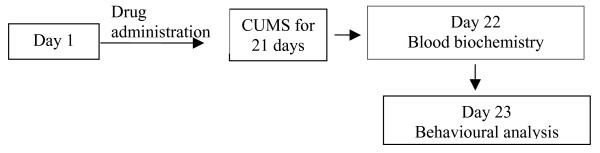
Schematic diagram representing the study design.

Four groups of rats were exposed to CMS and received p.o.: vehicle (0.3% Carboxy methyl cellulose CMC, 1 ml/kg), diazepam (DZM, 2 mg/kg), CS (125 and 250 mg/kg) respectively. Drugs (CS and DZM) were prepared as fine suspension in 0.3% CMC and administered one hour before the stress exposure for 21 days (Figure 1).

### Induction of stress

Stress was induced by employing the Chronic Unpredictable Mild Stress (CMS) protocol. It was based on the modus operandi originally used by Pal and Dandiya (1993) [19] with minor modifications. Each stress regimen was carried out for 2 periods with the following stressors: food deprivation for 24 h, day-night reversal, soiled bedding (~150 ml water per cage) for 22 h, cage tilting (~45 degree inclined) for 22 h, crowded housing (10 animals per cage) for 12 h, exposure to a novel odor (household air freshener) for 12 h, restraint stress for 20 min, cold stress 4–8°C and heat stress 38–39°C for 20 min and intermittent white noise (80 dB) for 5 h for 3 periods.

### Behavioural analysis

#### Open field exploratory behaviour test

Open field test was used to study the exploratory and anxiety behaviour of rats [20]. The open field apparatus consisted of a square arena 60 × 60 cm with 40 cm high wall. The entire apparatus was painted black except for 6 mm white lines that divided the floor into 16 equal size squares. The apparatus was illuminated with a low intensity diffuse light (45 W) situated 45 cm above the floor level. Entire room, except the open field was kept dark during the experiment. Each animal was placed in the central square and observed for 5 min and the following behaviours were recorded. Ambulation – the number of grid lines it crossed with all the four paws; rearing – by counting the number of times the animal stood on its hind limbs; grooming-number of times the animal made these responses viz. grooming of the face, licking/cleaning and scratching the various parts of the body, defecation – the number of fecal boli excreted during the period and immobility period. Between tests, the apparatus was cleaned with 5% alcohol.

#### Behavioural despair test

Behaviour despair model used by Porsolt et al., 1978 was followed to test antidepressant activity [21]. Rats were forced to swim individually in a glass jar (45 × 12 × 45 cm^3^) containing fresh water of height 35 cm and maintained at 25 ± 3°C. After an initial 2–3 min period of vigorous activity, each animal assumed a typical immobile posture. A rat was considered to be immobile when it remained floating in the water without struggling, making only minimum movements of its limbs necessary to keep its head above water. The total duration of immobility was recorded during the next 4 min of a total 6 min test. Rats were then allowed to dry in a pre-warmed enclosure (~32°C) before being returned to their home cage. All the behavioural experiments were carried out between 0900–1400 h.

#### Blood and organs collection

Twenty four hours after the last stress administration the blood was collected from overnight fasted rats through retro orbital puncture for biochemical analysis. Plasma was separated by centrifuging the blood at 4000 rpm for 10 min. Plasma glucose and total lipids were estimated using diagnostic kits immediately (Ecoline, Merck, India).

During conditions of stress, kidney synthesizes ascorbic acid to compensate glutathione loss. Hence, the endogenous antioxidants such as SOD, CAT; non-enzymic antioxidant – ascorbic acid and TBARS levels in were studied in brain, kidneys and adrenals. On 23^rd ^day rats were sacrificed and organs were isolated, weighed and homogenized immediately with ice cold 10% KCl. The supernatant was separated by centrifugation at 5000 rpm for 10 min and stored at -80°C until the assay was done.

#### Superoxide dismutase (SOD)

SOD estimation was performed based on its ability to spontaneously inhibit oxidation of adrenaline to adrenochrome [22]. 2.78 ml of sodium carbonate buffer (0.05 mM; pH 10.2), 100 μl of EDTA (1.0 mM) and 20 μl of the supernatant or sucrose (blank) were incubated at 30°C for 45 min. Thereafter, the reaction was initiated by adding 100 μl of adrenaline solution (9.0 mM). The change in the absorbance was recorded at 480 nm for 8 min. Throughout the assay procedure temperature was maintained at 30°C. One unit of SOD produced approximately 50% of auto-oxidation of adrenaline. Results were expressed as Units/mg protein.

#### Catalase (CAT)

CAT measurement was done based on its ability to decompose hydrogen peroxide (H_2_O_2_) [23]. Briefly, 2.25 ml of potassium phosphate buffer (65 mM, pH 7.8) and 100 μl of the supernatant or sucrose (0.32 M) were incubated at 25°C for 30 min. H_2_O_2 _(7.5 mM; 650 μl) was added to initiate the reaction. The change in absorbance was measured for 3 min at 240 nm and the results were expressed as U/mg protein.

#### Ascorbic acid

Ascorbic acid was estimated by spectroscopy method [24]. One ml of 4% trichloroacetic acid was added to 250 μl of supernatant. This mixture was kept at 4°C for 1 h and centrifuged at 4000 rpm for 10 min. A 0.5 ml aliquot of the supernatant was taken and 125 μl of dinitrophenyl hydrazine was added. This mixture was heated in water bath at 85°C for 30 min. Then 875 μl of 65% sulphuric acid was added slowly under ice cold water. After complete mixing, the tubes were allowed to stand for 30 min at room temperature, and the OD was read at 540 nm. The calibration curve was prepared by using ultrapure ascorbic acid as standard (1 mg/ml in sodium acetate buffer). The blank and the standard solution were processed similarly. The ascorbic acid levels were expressed as μg/mg of adrenals.

#### Lipid peroxide

The lipid peroxidation in terms of thiobarbituric acid reactive substances (TBARS) was measured using the method of Ohkawa et al., (1979) [25]. Mixture of 500 μl of the supernatant, 200 μl of 8% sodium dodecyl sulphate, 1.5 ml of 20% acetic acid solution, 1.5 ml of 0.9% aqueous solution of thiobarbituric acid and 1.3 ml of distilled water were heated in boiling water bath for 30 min. After cooling, the red chromogen was extracted into 5 ml mixture of n-butanol and pyridine (15:1v/v). The organic layer was separated and the absorbance was measured at 532 nm. Lipid peroxide content was expressed as nmole of malondialdehyde (MDA)/mg tissue. The calibration curve was prepared by using 1, 1, 3, 3-tetra ethoxypropane (TEP) as standard.

#### Protein estimation

Protein content of the samples was estimated by the method of Lowry et al., using bovine serum albumin as standard [26].

### Statistical analysis

Results were expressed as mean ± SE. Data were analyzed by one-way ANOVA followed by Dunnet's multiple comparisons using GraphPad Prism 4.0, statistics software. In all the tests, the criterion for statistical significance was p < 0.05.

## Results

### Phenolic content

Total phenolic content of 60% methanolic extract of CS was found to be 8.54 ± 0.16 %w/w.

### Body weight

Body weight of experimental animals was measured on day 1 and 22 of the CMS period. The 21 days exposure to CMS significantly decreased the body weight (4%) [F(4,25) = 2.170, p < 0.05] in comparison to the control group (37%). Treatment with CS 125 (17%) and 250 mg/kg (15%)or DZM (31%) augmented the weight increase of the rats however did not reverse completely the weight change induced by CMS (Table 1). Gross pathological examination of the CMS rats revealed, the presence of gastric ulcer (+++). Treatment with CS 125 mg/kg (++) and 250 mg/kg (++) decreased the ulcer score. Similar findings were observed with DZM treated group (+). The results indicated the protective ability of these drugs against CMS induced ulceration. Gastric ulcer scoring was performed as described earlier by Sanyal et al (1982) [27].

**Table 1:** Effect of CS on body weight, glucose and total lipids level in stressed rats

**Groups**	**Body weight (g)**		**Glucose (mg/dl)**	**Total lipids (mg/dl)**
	
	**Day 1**	**Day 22**		
**Control**	167.0 ± 10.7	226.2 ± 14.3	77.5 ± 3.8	693.33 ± 24.9
**CMS (0.3%CMC, 1 ml/kg)**	169.2 ± 9.8	176.7 ± 8.43*	132.5 ± 11.3**	930.33 ± 33.0**
**CMS+DZM (2 mg/kg, p.o)**	170.8 ± 10.7	215.5 ± 15.5	55.50 ± 7.2^††^	715.52 ± 29.1^††^
**CMS+CS (125 mg/kg, p.o)**	165.5 ± 11.3	197.3 ± 12.7	83.69 ± 4.6^††^	583.90 ± 10.6^††^
**CMS+CS (250 mg/kg, p.o)**	171.3 ± 11.4	199.7 ± 12.1	61.75 ± 8.0^††^	619.08 ± 32.7^††^

Data are expressed as mean ± SEM. * and † indicates statistical significance in comparison to control and stress groups respectively and denotes *, † p < 0.05 and **, † † p < 0.01

### Plasma biochemistry

In comparison to the control rats a significant [F(4,25) = 16.50, p < 0.05] increase in fasting blood glucose and total lipid levels was observed in CMS group. CS controlled the glucose and total lipid alterations significantly [F(4,25) = 24.42, p < 0.01] and the results were comparable with that of the reference drug DZM (Table 1).

### Behaviour analysis

#### Open field exploratory behaviour test

CMS rats exhibited anxious behaviour as evidenced by decreased ambulation [F(4,25) = 6.108, p < 0.05], rearing [F(4,25) = 6.729, p < 0.01], increased grooming [F(4,25) = 7.739, p < 0.01] and immobility period [F(4,25) = 56.49, p < 0.01] in comparison to control rats. Treatment with CS significantly reversed the stress induced behavioural alteration in a dose dependent manner as observed by increased ambulation, rearing and decreased immobility period as compared to the CMS group. Results were comparable with that of the reference drug DZM. In the open field exploration the CMS rats evinced significant decrease in the central activity in comparison to control rats. DZM increased the central activity in comparison to CMS group. Though the CS treated rats exhibited an increase in central action it was not significant (Table 2).

**Table 2:** Effect of CS on open field exploratory test and behavioural despair test in CMS rats

	**Open field exploratory test**						**Behavioural despair test**
	
**Groups**	**Total**	**Ambulation Central**	**Ratio**	**Rearing**	**Grooming**	**Immobility period (sec)**	**Immobility period (sec)**
**Control**	75.8 ± 3.6	11.5 ± 0.44	0.1575	22.33 ± 4.10	6.67 ± 1.09	48.16 ± 4.2	72.33 ± 7.1
**CMS (0.3% CMC 1 ml/kg)**	51.7 ± 2.5**	3.83 ± 0.42*	0.0740	8.83 ± 1.63**	11.5 ± 0.96**	197.83 ± 15.03**	126.33 ± 11.2**
**CMS+DZM (2 mg/kg)**	68.3 ± 3.78^†^	9.17 ± 0.27^†^	0.1341	14.00 ± 2.1	4.2 ± 0.9^††^	36.00 ± 4.69^††^	56.17 ± 6.1^††^
**CMS+CS (125 mg/kg)**	71.8 ± 6.5^†^	8.33 ± 2.06	0.1160	15.5 ± 1.69	7.5 ± 0.76^†^	79.5 ± 9.9^††^	123.3 ± 10.7
**CMS+CS (250 mg/kg)**	80.0 ± 4.6^††^	7.33 ± 2.16	0.0916	23.83 ± 1.30^††^	6.33 ± 1.06^††^	49.0 ± 5.45^††^	120.16 ± 9.7

Data are expressed as mean ± SEM. * and † indicates statistical significance in comparison to control and stress groups respectively and denotes *, † p < 0.05 and **, † † p < 0.01

#### Behavioural despair test

Results of the behavioural despair test are represented in Table 2. A significant [F(4,25) = 12.83, p < 0.05] increase in immobility period was observed in CMS group in comparison to control rats indicating the presence of depression in the former group. Treatment with diazepam significantly (p < 0.01) attenuated the stress induced depression as recorded by decreased immobility period whereas CS failed to alter the stress induced depressive behaviour.

### Antioxidant evaluation

#### Superoxide dismutase

Application of different stressors for 21 days significantly depleted SOD activity in brain [F(4,25) = 110.9, p < 0.05], kidneys [F(4,25) = 10.69, p < 0.05] and adrenals [F(4,25) = 4.743, p < 0.05] of CMS rats. CS (125 and 250 mg/kg), significantly improved the SOD activity in all the tissues in a dose dependent manner. Diazepam improved the SOD level only in kidney tissue but failed to show protection in adrenals and brain (Table 3).

**Table 3:** Effect of CS on endogenous antioxidant and TBARS level in CMS rats

	**Groups**				
	
**Parameters**	**Control**	**CMS**	**CMS+DZM (2 mg/kg, p.o)**	**CMS+CS**	
				
				**125 mg/kg, p.o**	**250 mg/kg, p.o**
**SOD (U/mg protein)**					
Brain	1.09 ± 0.03	0.21 ± 0.02**	0.98 ± 0.04^††^	1.30 ± 0.07^††^	1.54 ± 0.06^††^
Kidneys	0.75 ± 0.03	0.51 ± 0.05**	0.66 ± 0.05	0.71 ± 0.05^††^	0.88 ± 0.01^††^
Adrenals	0.99 ± 0.10	0.56 ± 0.04**	0.85 ± 0.07^†^	0.92 ± 0.09^†^	0.83 ± 0.06
					
**CAT (U/mg protein)**					
Brain	1.77 ± 0.13	1.67 ± 0.28	1.50 ± 0.36	2.13 ± 0.41	2.63 ± 0.56
Kidneys	2.87 ± 0.35	1.43 ± 0.37*	1.83 ± 0.40	3.14 ± 0.24^††^	3.93 ± 0.14^††^
Adrenals	1.27 ± 0.15	0.25 ± 0.02**	0.8 ± 0.06^††^	0.41 ± 0.08	0.72 ± 0.09^††^
					
**Ascorbic acid (μg/mg protein)**					
Brain	13.75 ± 3.50	14.25 ± 3.50	12.50 ± 3.13	10.38 ± 2.63	12.25 ± 3.13
Kidneys	17.83 ± 2.00	23.33 ± 1.67*	18.33 ± 1.50	20.50 ± 1.17	18.33 ± 0.67
Adrenals	43.33 ± 4.7	90.00 ± 11.33**	49.67 ± 6.53^††^	58.67 ± 9.13^†^	68.67 ± 6.67
					
**TBARS (nmol of MDA/mg tissue)**					
Brain	0.15 ± 0.02	0.89 ± 0.12**	0.42 ± 0.10^††^	0.48 ± 0.07^††^	0.38 ± 0.09^††^
Kidneys	0.19 ± 0.02	0.84 ± 0.07**	0.53 ± 0.05	0.58 ± 0.04	0.38 ± 0.19^††^
Adrenals	0.28 ± 0.24	1.23 ± 0.13**	1.04 ± 0.21^†^	1.32 ± 0.28	1.09 ± 0.33^†^

Data are expressed as mean ± SEM. * and ^† ^indicates statistical significance in comparison to control and stress groups respectively and denotes *, † p < 0.05 and **, † † p < 0.01

#### Catalase

In comparison to control rats a significant decrease in the CAT activity was observed in kidney [F(4,25) = 10.29, p < 0.05] and adrenals [F(4,25) = 18.95, p < 0.05] of CMS rats. But brain CAT level remains unaltered. CS did not demonstrate any significant increase in brain CAT level. It produced a significant (p < 0.01) increase in CAT level in kidneys at both doses. The CAT level in adrenals was found to increase significantly (p < 0.01) only in the group treated with 250 mg/kg of CS (Table 3). DZM recovered CAT activity significantly in adrenals and failed to produce any such effect in brain and kidney.

#### Ascorbic acid

Significant [F(4,25) = 5.222, p < 0.05] elevation in ascorbic acid levels were observed in the adrenals of CMS rats. No alteration in kidney and brain ascorbic acid level was observed. Treatment with CS during stress period significantly decreased the adrenal ascorbic acid levels. However the ascorbic acid levels in kidneys and brain did not show any significant observation with CS treatment (Table 3). DZM decreased ascorbic acid level in adrenal tissues of CMS rats but no marked effect was noted in brain and kidneys.

#### Thiobarbituric acid reactive substances

Significant elevation of TBARS content in brain [F(4,25) = 9.567, p < 0.01], kidney [F(4,25) = 6.41, p < 0.01] and adrenals [F(4,25) = 11.54, p < 0.01] was observed in CMS rats in comparison to control rats. Administration of DZM controlled this elevation significantly (p < 0.05) in all the tissues studied. High dose of CS significantly decreased the TBARS content in kidneys and adrenal tissues but not in brain tissue (Table 3).

## Discussion

Long-term exposure to multiple stressors can cause depression in humans. Induction of depression using CMS is considered as the most congruent animal model of depressive conditions observed in humans after long term exposure to multiple stressors [28]. CMS is a well-validated model, which has predictive, constructive and face validity [29]. The results of the current study demonstrated decreased body weight along with elevated blood glucose and total lipid levels in rats exposed to CMS, which are reminiscent of the features of subgroups of clinically depressed patients [30,31]. The results were found to be consistent with the report of glucose intolerance in rats subjected to CMS [15]. The Brain-Pancreas Relative Protein (BPRP) was reported to induce depression and its loss resulted in decrease blood level of insulin leading to increase blood glucose level in CMS model [32]. Administration of CS during stress period reversed the metabolic changes indicating certain influence on neuro-endocrinological system.

Open field exploratory test is commonly used to assess locomotor, exploratory and anxiety-like behaviour in experimental animals (rats/mice). In the open field test, CMS rats exhibited decreased ambulation and rearing which indicated reduced exploration and apathy respectively in these animals. Increased immobility period and grooming activity depicted a higher level of anxiety [15]. Treatment with CS reversed the altered open field behaviour significantly and this probably is due to its anxiolytic effect. Behavioural despair test is used to measure the antidepressant effect of drugs. CMS rats exhibited depression evidenced by increased immobility period in behavioural despair test. Administration of CS failed to show any effect on the immobility period in CMS rats. The results indicated that CS possesses anxiolytic activity but no antidepressant activity. The standard drug diazepam controlled the metabolic alterations and gastric ulcers in CMS rats indicate the GABAergic role in CMS induced physiological alterations. Earlier reports indicated that diazepam reversed the stress induced non-grooming and grooming behaviour and gastric ulcers in rats [33,34] which supports our present findings. Further, diazepam reversed the CMS induced behavioural alterations and showed anxiolytic activity in open field test, antidepressant activity in behavioural despair test. This effect of diazepam may be attributed to its anti stress property. Hence, it can be speculated that CS might exert similar activity on GABAergic system rather than the adrenergic or serotonergic system former plays a vital role in anxiolytic property.

Antioxidant study in different tissues of CMS rats revealed decreased SOD, CAT levels and increased TBARS levels denoting that administration of different stressors triggered free radicals generation. Further, adrenal ascorbic acid level was found to be elevated in stressed rats which clearly denoted that CMS rats were under oxidative stress and the biological system exhibits an adaptive mechanism. In stressed conditions similar metabolic alterations were reported resulting in the imbalance of energy utilization and consequently in the generation of superoxide anion, hydrogen peroxide and hydroxyl radical ions as the major reactive oxygen species (ROS) in the bio-systems which provoked cell lipid membrane peroxidation. Lipid peroxide products reportedly caused widespread cellular injury [35]. Endogenous enzymic antioxidants such as SOD, which dismutases the highly reactive superoxide anion to the less reactive species H_2_O_2 _[36], CAT, a haeme containing enzyme, which scavenges hydrogen peroxide to water and molecular oxygen [37] and non-enzymic ascorbic acid, which is a water-soluble antioxidant forage free radical protect the biological system from oxidative stress [38].

Treatment with CS decreases the CMS-induced SOD and CAT alterations. Further, CS treatment led to decreased TBARs content in different tissues, resulting in attenuation of the adverse effects of chronic stress due to generation of ROS. The phenolic content of CS was found to be 8.54% which may contribute to the observed antioxidant effect. The active principles flavone 6"-*O *acetyl scoparin [10], flavonals, namely, rutin, quercetin, quercitrin, iso rhamnetin and kaempferol [11] and isoflavones like genistein and sarothamnoside [12] might have contributed to the antioxidant property of the herb. Earlier reports demonstrated that herbs containing flavonoids, tannins and phenolic compounds were found to possess antioxidant property and attenuate cell death induced by oxidative stress [39,40] which supported our present findings. Results of the present study indicate that the anti-stress activity of CS may be due to its antioxidant potential.

## Conclusion

To conclude, CS showed moderate anxiolytic activity and high propensity to improve the antioxidant status in CMS rats. Hence, this antioxidant effect may be the cardinal mechanism that is expressed as the adaptogenic activity of CS. Further studies may elucidate the possibility of the commercial use of CS for the benefit of patients suffering from psychotic disorders.

## Competing interests

The authors declare that they have no competing interests.

## Authors' contributions

JN: Performed the study. CSB: Performed the study and manuscript drafting. TH: Helped to perform biochemical analysis. MR: Study design, data analysis, manuscript drafting and supervised the study.

## Pre-publication history

The pre-publication history for this paper can be accessed here:


